# Pharmacodynamics of cisplatin in human head and neck cancer: correlation between platinum content, DNA adduct levels and drug sensitivity in vitro and in vivo

**DOI:** 10.1038/sj.bjc.6690015

**Published:** 1999-09-24

**Authors:** M J P Welters, A M J Fichtinger-Schepman, R A Baan, A J Jacobs-Bergmans, A Kegel, W J F van der Vijgh, B J M Braakhuis

**Affiliations:** Toxicology Division, TNO Nutrition and Food Research Institute, PO Box 360, AJ Zeist, 3700, The Netherlands; Klinisch Dierexperimenteel Laboratorium, Vrije Universiteit, Amsterdam,1007 MB, The Netherlands; Department of Medical Oncology, Vrije Universiteit, PO Box 7057, Amsterdam,1007 MB, The Netherlands; Department of Otolaryngology, Vrije Universiteit, PO Box 7057, Amsterdam, 1007 MB, The Netherlands

**Keywords:** cisplatin, DNA adducts, head and neck cancer, platinum accumulation, cisplatin sensitivity

## Abstract

Total platinum contents and cisplatin–DNA adduct levels were determined in vivo in xenografted tumour tissues in mice and in vitro in cultured tumour cells of head and neck squamous cell carcinoma (HNSCC), and correlated with sensitivity to cisplatin. In vivo, a panel of five HNSCC tumour lines growing as xenografts in nude mice was used. In vitro, the panel consisted of five HNSCC cell lines, of which four had an in vivo equivalent. Sensitivity to cisplatin varied three- to sevenfold among cell lines and tumours respectively. However, the ranking of the sensitivities of the tumour lines (in vivo), also after reinjection of the cultured tumour cells, did not coincide with that of the corresponding cell lines, which showed that cell culture systems are not representative for the in vivo situation. Both in vitro and in vivo, however, significant correlations were found between total platinum levels, measured by atomic absorption spectrophotometry (AAS), and tumour response to cisplatin therapy at all time points tested. The levels of the two major cisplatin–DNA adduct types were determined by a recently developed and improved^32^P post-labelling assay at various time points after cisplatin treatment. Evidence is presented that the platinum–AG adduct, in which platinum is bound to guanine and an adjacent adenine, may be the cytotoxic lesion because a significant correlation was found between the platinum–AG levels and the sensitivities in our panel of HNSCC, in vitro as well as in vivo. This correlation with the platinum–AG levels was established at 1 h (in vitro) and 3 h (in vivo) after the start of the cisplatin treatment, which emphasizes the importance of early sampling. © 1999 Cancer Research Campaign


British Journal of Cancer (1999) **79**(1), 82–88
© 1999 Cancer Research Campaign

### Pharmacodynamics of cisplatin in human head and neck cancer: correlation between platinum content, DNA adduct levels and drug sensitivity in vitro and in vivo

**MJP Welters[1], AMJ Fichtinger-Schepman[1], RA Baan[1], AJ Jacobs-Bergmans[1], A Kegel[2], WJF van der Vijgh[3] and BJM Braakhuis[4]**

[1]Toxicology Division, TNO Nutrition and Food Research Institute, PO Box 360, 3700 AJ Zeist, The Netherlands; [2]Klinisch Dierexperimenteel Laboratorium and Departments of [3]Medical Oncology and [4]Otolaryngology, Vrije Universiteit, PO Box 7057, 1007 MB Amsterdam, The Netherlands

**Summary** Total platinum contents and cisplatin–DNA adduct levels were determined in vivo in xenografted tumour tissues in mice and in vitro in cultured tumour cells of head and neck squamous cell carcinoma (HNSCC), and correlated with sensitivity to cisplatin. In vivo, a panel of five HNSCC tumour lines growing as xenografts in nude mice was used. In vitro, the panel consisted of five HNSCC cell lines, of which four had an in vivo equivalent. Sensitivity to cisplatin varied three- to sevenfold among cell lines and tumours respectively. However, the ranking of the sensitivities of the tumour lines (in vivo), also after reinjection of the cultured tumour cells, did not coincide with that of the corresponding cell lines, which showed that cell culture systems are not representative for the in vivo situation. Both in vitro and in vivo, however, significant correlations were found between total platinum levels, measured by atomic absorption spectrophotometry (AAS), and tumour response to cisplatin therapy at all time points tested. The levels of the two major cisplatin–DNA adduct types were determined by a recently developed and improved $^{32}P$ post-labelling assay at various time points after cisplatin treatment. Evidence is presented that the platinum–AG adduct, in which platinum is bound to guanine and an adjacent adenine, may be the cytotoxic lesion because a significant correlation was found between the platinum–AG levels and the sensitivities in our panel of HNSCC, in vitro as well as in vivo. This correlation with the platinum–AG levels was established at 1 h (in vitro) and 3 h (in vivo) after the start of the cisplatin treatment, which emphasizes the importance of early sampling.

**Keywords:** cisplatin; DNA adducts; head and neck cancer; platinum accumulation; cisplatin sensitivity

Cisplatin is widely used for the treatment of various solid tumours. However, the response to this drug varies not only between tumour types, but also among tumours of a certain type. It is generally believed that the anti-tumour effect of cisplatin is due to direct binding of the drug to DNA, resulting in platinum–DNA monoadducts and various bifunctional adducts (Fichtinger-Schepman et al, 1985; Eastman, 1986). The main adduct formed is *cis*-platinum diammine-d(GpG) (abbreviated platinum-GG), with platinum bound to two adjacent guanines. Another major intrastrand crosslink is *cis*-platinum diammine-d(ApG) (platinum-AG), in which platinum is bound to guanine and an adjacent adenine. The other bifunctional adducts are *cis*-platinum diammine-$(dG)_2$ (G-platinum-G), the intrastrand adducts in which platinum is bound to two guanines separated by one or more other nucleotide(s), and the interstrand crosslinks between two guanines in opposite strands. These adducts can inhibit cell proliferation and, if not repaired or tolerated, finally lead to cell death.

In patients with the same type of tumour, a fairly large inter-individual variation in DNA adduct levels, measured in white blood cells (WBCs), has been observed (Fichtinger-Schepman et al, 1990; Parker et al, 1993; Poirier et al, 1993; Reed et al, 1988, 1993; Schellens et al, 1996). These clinical investigations provided evidence that higher adduct levels correlate with a better clinical outcome. In contrast to these findings, there are also reports showing no correlation between adduct levels in WBCs and the efficacy of therapy (Gupta-Burt et al, 1993; Motzer et al, 1994; Fisch et al, 1996). All these clinical studies, however, were performed in patients with a variety of solid tumours and after different treatment regimens, which could be a reason for the inconsistency of the results. Furthermore, blood samples were collected at different time points, although in most studies this was done at 24 h after cisplatin administration. In addition, the methods used for adduct detection and statistical analysis [using either the highest (peak) level or the mean value over several courses] were different between the various studies.

The basic question in all these investigations, however, is whether or not platinum-DNA adduct levels in the tumours correlate with the response to the cisplatin treatment. Because it is not feasible to obtain tumour biopsies from patients for such studies, the readily obtainable WBCs were used in most investigations as a substitute for the target tissue. In the present study, both the total platinum content and the platinum–GG and platinum–AG adduct levels were determined in human head and neck squamous cell carcinomas (HNSCC) grown in nude mice and as cultured cells, at various time points after the start of cisplatin treatment. Responses of the tumours to this treatment were correlated with both total platinum contents and these platinum–DNA adduct levels. Because the pharmacokinetics of cisplatin are similar in nude mice and humans (Kubota et al, 1993), this study will provide more insight into factors involved in the clinical response of patients to cisplatin.

*Received 18 November 1997*
*Revised 8 March 1998*
*Accepted 21 May 1998*

*Correspondence to:* BJM Braakhuis, Department of Otolaryngology, University Hospital Vrije Universiteit, PO Box 7057, 1007 MB, Amsterdam, The Netherlands

### MATERIALS AND METHODS

### Tumour lines

Human head and neck squamous cell carcinomas (HNSCC) were used in two model systems.

*In vitro*

Cell lines UM-SCC-14C (abbreviated as 14C), UM-SCC-22B (22B) and UM-SCC-35 (35) were established directly from the tumour biopsy of the patient (Carey et al, 1990). VU-SCC-OE (OE) and VU-SCC-RO (RO) were established from xenografts that were initiated originally by transplanting tumour fragments from patients into mice (Welters et al, 1997*a*). Cells were cultured routinely in Dulbecco's modified Eagle medium [Life Technologies (Gibco BRL), Breda, The Netherlands] supplemented with 5% heat-inactivated fetal calf serum (Flow, Irvine, UK), 50 U $ml^{-1}$ penicillin and 50 μg $ml^{-1}$ streptomycin (Life Technologies).

*In vivo*

Xenografts were established on female, athymic, nude-nu mice (6–8 weeks old; Harlan, Zeist, The Netherlands) either by subcutaneous injection of cultured tumour cells (14C and 22B) in suspension at both sides or by implantation of tissue fragments of the tumour from the patient (OE, RO and HN). Upon growth, the tumour lines were maintained by serial transplantation (Braakhuis et al, 1995). Tumour volumes ($mm^3$) were determined twice weekly by use of a vernier calliper and calculated according to the formula: $0.5 \times \text{length} \times \text{width} \times \text{height}$. This method of measuring tumour volumes has proved to be most accurate (Tomayko and Reynolds, 1989). Animal experiments were performed according to the Dutch law on animal experiments and after approval of the appropriate ethics committees at our institutes.

### DNA index

Cultured HNSCC cells were trypsinized and harvested. Tumour tissues were dissociated by use of trypsin for 30 min at 37°C, followed by filtration through 100-μm nylon filters in order to obtain single cells. The DNA of these cells was labelled with the fluorescent DNA intercalator propidium iodide as described earlier (Welters et al, 1997*a*) and the DNA index was determined by flow cytometry.

### Sensitivity to cisplatin

Tumour cells in culture and tumour-bearing mice were treated with cisplatin (Platinol, Bristol-Myers Squibb, Weesp, The Netherlands) to determine the growth response and to obtain samples for the analysis of total platinum and platinum–DNA adduct levels.

*In vitro*

The cisplatin-induced inhibition of cell proliferation, as a measure of the sensitivity to this drug, has been determined and published previously (Welters et al, 1997*a*). The HNSCC cells were exposed to various concentrations of cisplatin for 72 h at 37°C and the inhibition of cell growth was determined colorimetrically with the sulphorhodamin B assay (SRB). The cisplatin sensitivity was expressed as $IC_{50}$ value, i.e. the concentration of the drug causing 50% growth inhibition compared with untreated control cells.

*In vivo*

Mice bearing HNSCC tumours with a volume between 50 and 200 $mm^3$ were randomly divided into treatment and control groups, with 6–10 tumours per group. Cisplatin (7 mg $kg^{-1}$) was given intravenously, twice with a 7-day interval. The drug was administered at the maximum tolerated dose, i.e. the dose leading to a weight loss of no more than 5–15%. Anti-tumour activity was expressed as growth delay factor (GDF), defined as the difference between the mean values of the time required by tumours of treated and control animals to double their volume, divided by the mean value of the tumour doubling time in control mice. A second method to quantify the therapeutic results of the cisplatin treatment was by determination of the treated/control value (T/C), i.e. the mean tumour volumes after treatment divided by the tumour volumes at the start of the treatment. The lowest value of this ratio at any time point during the observation period was considered as optimal T/C value. Complete responses were scored when a tumour regressed and did not show regrowth for a period of 3 months.

### Intracellular cisplatin

The levels of total platinum were determined both in cultured cells and in xenografts of cisplatin-treated mice.

*In vitro*

Cells were treated continuously for 1, 6, 24, 48 or 72 h with 10 μM cisplatin, whereafter the cells were washed and harvested. Portions of the samples were processed to determine the cellular platinum content by atomic absorption spectroscopy (AAS) (Spectra AA-300 Zeeman AAS, Varian, Houten, The Netherlands), as described previously (Welters et al, 1997*a*).

*In vivo*

Tumour-bearing mice were treated twice with cisplatin as indicated above and sacrificed at various time intervals after the second injection (3 h, 24 h, and 3, 7 and 14 days). The tumours were removed, quickly frozen in liquid nitrogen and stored at –80°C. One half of each tumour was prepared for measurement of total platinum levels by AAS according to the method of Siddik et al (1987). The tumour halves were weighed, lysed by addition of 0.5 ml of 1 M hyamine (benzethonium hydroxide, Sigma, St. Louis, MO, USA), and incubated overnight at 55°C. Platinum was measured after addition of 4.25 ml 0.2 M hydrochloric acid. Calibration standards and quality control samples of muscle tissue were spiked with cisplatin and treated in the same way as the samples (Korst et al, 1998).

### Platinum–DNA adducts

Portions of the cisplatin-treated cells and of the tumours obtained from treated mice were used for the analysis of the two DNA adducts platinum–GG and platinum–AG. Cell pellets and tumour tissues were lysed and DNA was isolated as described by Fichtinger-Schepman et al (1987, 1995*a*) and stored at –20°C until further analysis. After enzymatic digestion of the DNA, the adduct levels were determined by a novel, recently improved $^{32}P$ post-labelling assay after deplatination of the purified adducts (Welters et al, 1997*b*). The $^{32}P$-labelled products were separated by thin-layer chromatography and the radioactivity determined by

© Cancer Research Campaign 1999

phosphorimaging. The amounts of the two major intrastrand DNA adducts platinum–GG and platinum–AG were calculated on the basis of the labelling of a known amount of TpT, which was included as an internal standard.

### Statistical analysis

Correlations between the platinum content and DNA adduct levels with sensitivity to cisplatin were analysed by linear regression analysis. Only correlations with *P*-values of 0.05 or lower were considered to be significant. For the analysis, the area under the curve (AUC) was also utilized. AUC values (derived from plots of platinum content vs time or platinum–DNA adduct levels vs time) were calculated with the trapezoidal rule.

### RESULTS

In this study, the sensitivity to cisplatin in a panel of HNSCC xenografts (in vivo) was compared with that of the corresponding cultured cells (in vitro) and correlated with cisplatin accumulation and DNA adduct formation. As can be seen in Table 1, the cells growing as xenograft or in culture are comparable with respect to their DNA indices, with the exception of cell line RO. After several passages of RO, large cells containing more than one nucleus were observed, owing to the inability of the cells to complete their cell division. It was probably for this reason that RO cells could only be kept in culture for about 12 passages. Consequently, sufficient amounts of these cells to determine the cellular platinum and the platinum–DNA adduct levels could not be obtained. Unfortunately, we were not successful in establishing a cell line from the HN xenograft and no xenografts could be established from the UM-SCC-35 cell line. The DNA content of both the tumours and the cell lines was found to be rather consistent over time and during various passages.

### Sensitivity to cisplatin

The response of the tumours in the xenografted mice after cisplatin treatment are given in Table 2, as well as data on the tumour doubling times in the treated group and untreated controls. The 14C and HN xenografts were not sensitive. The best response, even some complete responses, were observed with RO. The intrinsic sensitivities to cisplatin of the cultured HNSCC cells were reported previously (Welters et al, 1997*a*). They are included in Table 2 to allow comparison with the in vivo data. $IC_{50}$ values ranged between 0.9 and 2.7 μM cisplatin.

Comparison of the ranking of the cisplatin sensitivities of the xenografts with those of the corresponding cultured cells revealed that both RO and OE differed in sensitivity when xenografted or cultured. These two tumour lines were established directly from the tumour biopsies obtained from the patient and the cell cultures were derived subsequently from the xenografts. In contrast, the 14C and 22B tumour lines were established after injection of the cultured cells into mice. To determine whether the difference between in vitro and in vivo responses of the HNSCC lines was due to the difference in the way the tumour lines were established, the cultured RO and OE cells were reinjected subcutaneously into mice and, after growth to tumours, the mice were treated with cisplatin. The responses to cisplatin of these newly established tumours were comparable to those of the original xenografts (data not shown).

**Table 1** DNA index of HNSCC xenografts and the corresponding cultured cells

| Tumour line | Degree of differentiation[a] | DNA index[b] | |
|---|---|---|---|
| | | Xenograft | Cell line |
| 14C | Poorly | 3.5 ± 0.1 | 3.1 ± 0.2 |
| 22B | Moderately | 2.3 ± 0.1 | 2.2 ± 0.5 |
| 35 | NA[c] | NA | 3.0 ± 0.1 |
| OE | Moderately | 3.3 ± 0.5 | 3.5 ± 0.3 |
| RO | Well | 1.8 ± 0.2 | 3.7 ± 0.2 |
| HN | Poorly/moderately | 3.1 ± 0.4 | NA |

[a]The xenografts were scored pathologically as described by Van Dongen et al (1989); [b]DNA index was determined by flow cytometry of the tumour cells derived from xenografts or from cell cultures. The DNA content appeared to be stable during several passages. The results are means ± s.d. of at least six independent measurements; [c]NA, not available.

**Table 2** Responses to cisplatin treatment in HNSCC xenografts and in cultured cells

| Tumour line | $IC_{50}$ value[a] | Tumour doubling time (days)[b] | | Tumour response[b] | |
|---|---|---|---|---|---|
| | | Control | Treated | GDF | T/C (%) |
| 14C | 2.7 ± 0.7 | 9.7 ± 1.6 | 11.6 ± 4.5 | 0.3 (–) | 69 (–) |
| 22B | 1.2 ± 0.3 | 10.2 ± 2.1 | 21.3 ± 6.0 | 1.1 (+) | 34 (+) |
| 35 | 0.9 ± 0.8 | NA | NA | NA | NA |
| OE | 2.3 ± 0.9 | 6.7 ± 1.8 | 12.9 ± 1.5 | 1.0 (+) | 34 (+) |
| RO | 2.4 ± 0.8 | 7.7 ± 2.0 | 22.8 ± 11.7 | 2.0 (++) | 19 (++) |
| HN | NA[c] | 6.1 ± 2.4 | 11.3 ± 5.4 | 0.9 (–) | 56 (–) |

[a]$IC_{50}$ value (in μM), the concentration of the drug causing 50% of growth inhibition, determined in a colorimetric (SRB) assay after exposure of the cells to cisplatin for 72 h (data from Welters et al, 1997*a*); [b]mice bearing human head and neck tumours were treated i.v. with 7 mg cisplatin $kg^{-1}$ twice with a 7-day interval. Responses to cisplatin therapy were determined in at least four independent experiments and expressed as GDF and T/C. GDF < 1 or T/C > 50 corresponds to a minimal effect (–); 1 < GDF < 2 or 25 < T/C < 50 to a moderate effect (+) and GDF > 2 or T/C < 25 to a strong effect with even some complete responses (++); [c]NA, not available.

### Intracellular cisplatin

As described previously for an in vitro study on a panel of seven HNSCC cell lines, most of which were also used in the present work, the cellular platinum levels correlated significantly with the $IC_{50}$ values, after incubation of the cells with 10 μM cisplatin during 1, 6, 24 or 48 h (Welters et al, 1997*a*). For these seven cell lines, positive correlations were found between sensitivity and the AUC values of the platinum vs time curves calculated from 1 h up to each time point studied during the total cisplatin incubation period of 48 h. The *r*-values were 0.83 ($P = 0.017$) when AUC was calculated over 6 h, 0.78 ($P = 0.031$) when calculated over 24 h and 0.78 ($P = 0.032$) over the 48-h time period. These correlations were found when data for cell line OE, derived from a patient previously treated with radiotherapy, were omitted (Welters et al, 1997*a*). In Figure 1, the cellular platinum levels as a function of the exposure time are given only for those cell lines for which the DNA adduct levels were also determined (see below).

To investigate whether the previously found correlations in vitro also exists in vivo, platinum levels were determined in the tumours at various time points after the second cisplatin treatment of the

© Cancer Research Campaign 1999

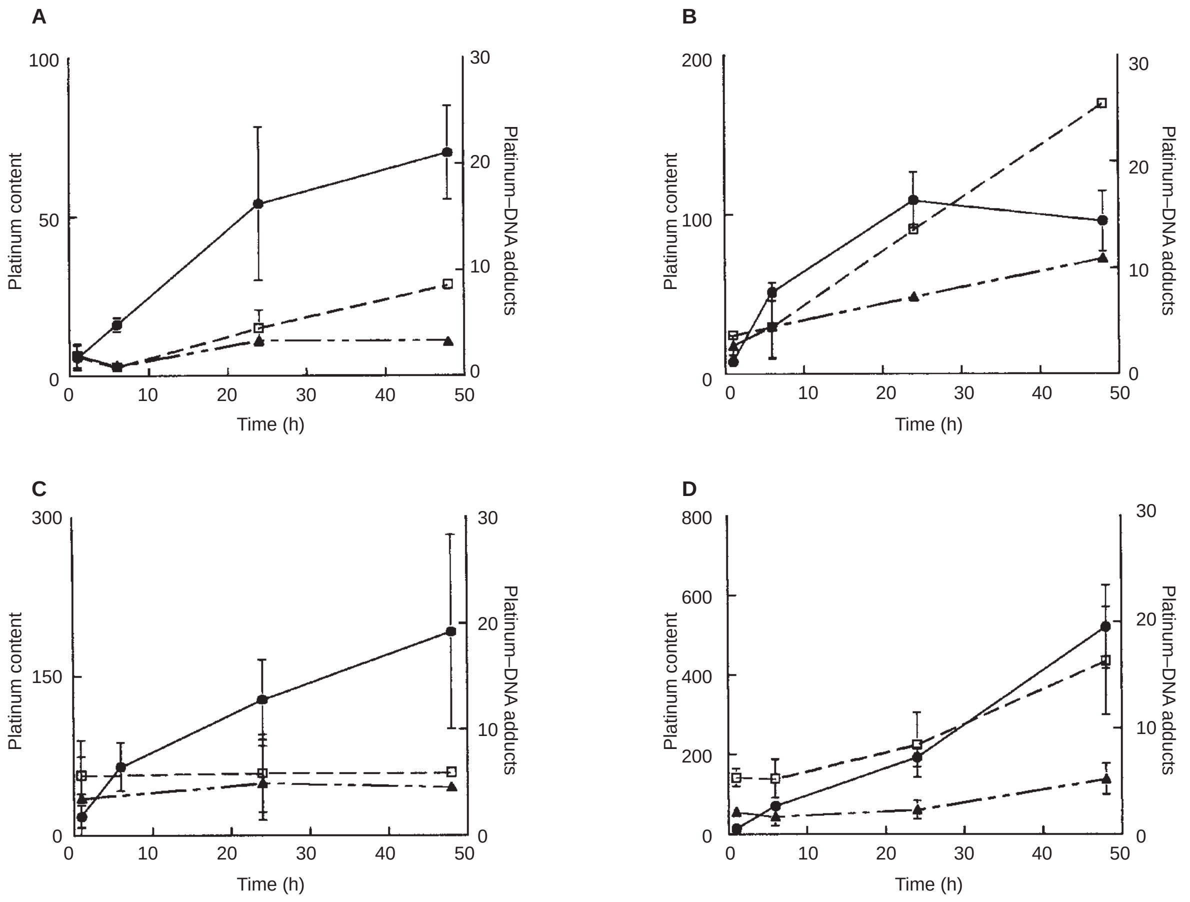

**Figure 1** Cultured human HNSCC cells 14C (**A**), 22B (**B**), 35 (**C**) and OE (**D**) were treated continuously with 10 μM cisplatin for the time period indicated. Total platinum content (pmol per $10^6$ cells; left *y*-axis) was measured by AAS (●; solid line). The two major intrastrand platinum–DNA adducts (number of adducts per $10^6$ nucleotides; right *y*-axis) were measured by the $^{32}P$ post-labelling assay (Welters et al, 1997*b*); platinum–GG (□; dashed line) and platinum–AG (▲; dashed line). Results are given of two independent experiments as means ± range of values tumour-bearing mice. As shown in Figure 2, 14C contained the lowest and RO the highest platinum levels. Comparison of the platinum levels with tumour response (expressed either as GDF or T/C; see Table 2) revealed no significant correlations at any time point measured. However, the AUC values of the platinum vs time curve, determined over the whole period of 14 days tested, showed a positive correlation with the GDF ($r = 0.95$, $P = 0.008$). When the period between 3 h and 3 days was taken as time interval for the AUC calculation, this correlation was even more significant ($r = 0.98$, $P = 0.0007$). Similarly, this AUC significantly correlated with the T/C values, showing an *r*-value of 0.86 ($P = 0.045$) when the AUC was calculated over 14 days and of 0.89 ($P = 0.028$) when calculated over the 3-day time period. Apparently, the amount of platinum present during the first time period after administration of cisplatin is very important for the efficacy of the treatment.

### Platinum–DNA adducts

The kinetics of cisplatin–DNA adduct formation and removal were studied in the cultured HNSCC cells as well as in the xenografts. The levels of the major adducts (platinum–GG and platinum–AG) were determined by $^{32}P$ post-labelling of digested DNA samples after deplatination of the adducts (Welters et al, 1997*b*). The in vitro results are depicted in Figure 1 and show that in three of the four HNSCC cell lines the adduct levels increased with time during the continuous exposure to cisplatin. No significant correlation could be found between the $IC_{50}$ values and either the adduct levels at the various time points or the AUC of platinum–DNA adduct levels calculated for the various time periods. The only exception, however, was the correlation with the platinum–AG adduct levels at 1 h after the start of cisplatin exposure ($r = -0.92$, $P = 0.045$).

In vivo, the platinum–GG and platinum–AG adduct levels were determined in portions of the same tumours that were used to obtain the total platinum content (Figure 2). It was found that the highest levels were reached at 3–24 h after the second injection with cisplatin, whereafter they decreased with time. The AUC calculated between 3 h and 14 days showed no correlation with the tumour response to cisplatin (expressed as GDF or T/C value). However, when the AUC was restricted to the period between 3 h and 3 days for both adducts, a relationship could be established. For platinum-GG, the correlation with GDF was not significant ($r = 0.82$, $P = 0.067$), but it was for the platinum–AG adduct levels ($r = 0.86$, $P = 0.042$). However, such a correlation was not found when adduct data were plotted against the T/C values. When the tumour responses were compared with the two adduct levels at the

© Cancer Research Campaign 1999

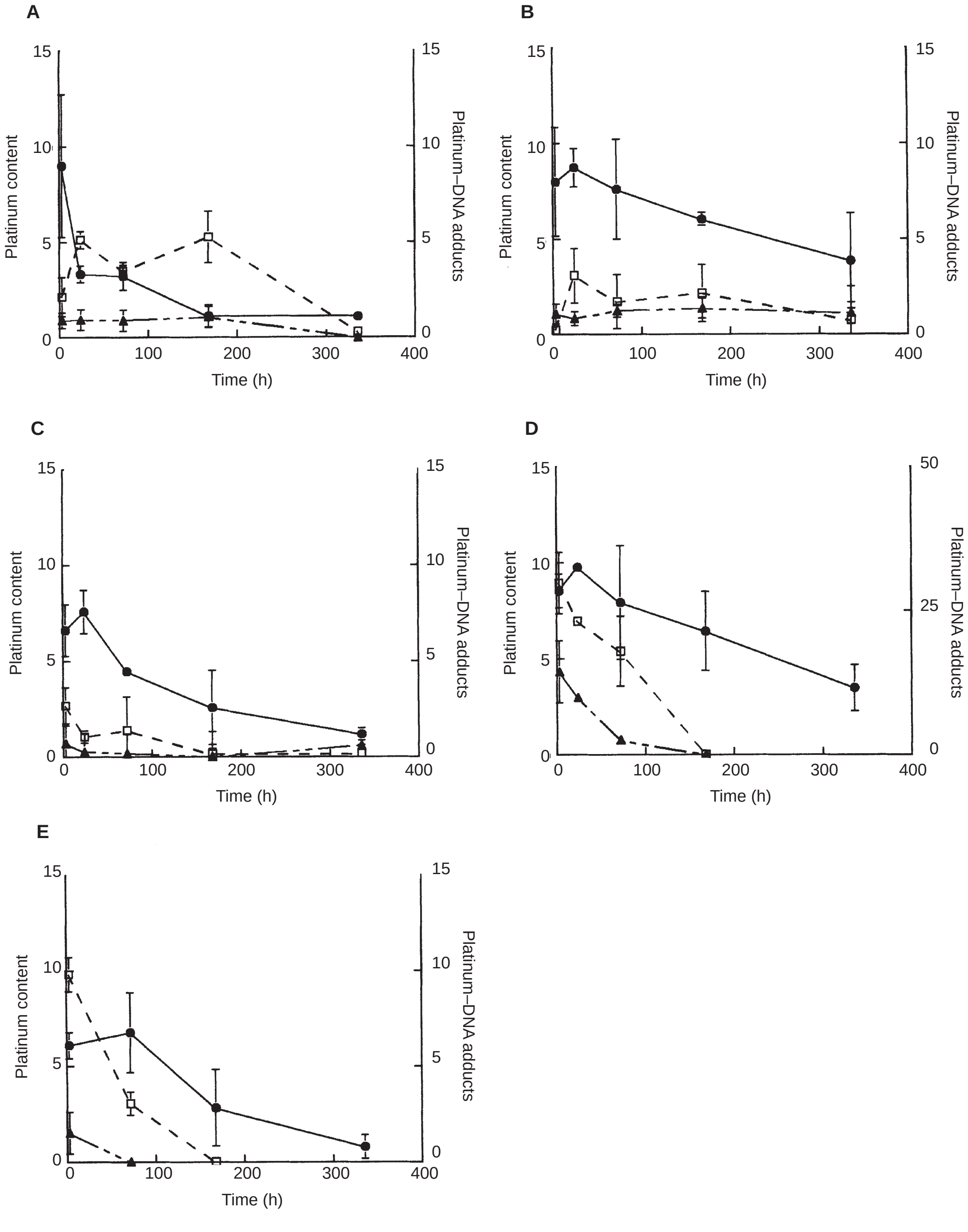

**Figure 2** Mice bearing the human HNSCC tumour 14C (**A**), 22B (**B**), OE (**C**), RO (**D**) or HN (**E**) were treated i.v. with 7 mg cisplatin $kg^{-1}$, twice with a 7-day interval. Tumours were removed at various time points after the second administration of the drug (3 h, 24 h, and 3, 7 and 14 days). Total platinum levels (fmol platinum $\mu g^{-1}$ tissue; left *y*-axis) in these tumours were determined by AAS (●; solid line) and the platinum–DNA adducts (number of adducts per $10^6$ nucleotides; right *y*-axis) were measured by the $^{32}P$ post-labelling assay (Welters et al, 1997*b*); platinum–GG (□; dashed line) and platinum–AG (▲; dashed line). Results are given of three independent experiments as means ± s.d.

various time points, a significant correlation was found only between GDF and the platinum–AG adducts at 3 h after cisplatin therapy ($r$ = 0.88, $P$ = 0.034). The platinum–AG data at 24 h did not correlate ($r$ = 0.86, $P$ = 0.096). The results suggest that sensitivity to cisplatin in HNSCC is determined by the amount of platinum–AG adduct that is present in the cell during a short time interval after cisplatin treatment.

### DISCUSSION

The relationship of responses to cisplatin treatment with total platinum content and cisplatin–DNA adduct levels was investigated in a panel of human HNSCC cultured cells and xenografts. A different ranking was observed when the responses of HNSCC to cisplatin were determined in vitro or in vivo, in particular with lines OE and

 *© Cancer Research Campaign 1999*

RO. These discrepancies could not be ascribed to genetic changes as a result of in vitro culturing because similar data were obtained when cell lines OE and RO were reinjected into mice. Also, the experiments measuring the DNA index did not provide evidence for such changes in the genetic material. The finding that tumours with the original drug–response properties were obtained after injection of cultured cells into mice was also published by Teicher et al (1990). This phenomenon clearly indicates that results obtained with cultured cells are not representative for the in vivo situation. In vivo, apparently more important factors are involved, for example vascularization. This difference between the in vitro and in vivo results may be eliminated when the cells are grown in a three-dimensional structure, e.g. as multicellular spheroids, as shown for tumour cells by Kobayashi et al (1993), or as post-confluent multilayered cultures, as shown by Pizao et al (1992).

In a comparative study with human ovarian carcinoma cell lines, Kelland et al (1992) investigated the in vitro and in vivo anti-tumour activity of cisplatin and three other platinum agents in eight companion lines. In agreement with our results, these authors did not find a correlation between in vitro SRB values and in vivo GDF data. However, they reported a significant correlation – for cisplatin only – between SRB results and tumour T/C values at 28 days, a time point which seems rather arbitrarily chosen. In our study, the lowest T/C ratio during the entire observation period was taken as optimal T/C value. Because of the rapid growth rate of the HNSCC xenografts, the 28-day time point could not be reached. It would be interesting to investigate, for example, platinum content and platinum–DNA adduct levels in the panel of ovarian carcinoma lines described by Kelland et al (1992), which span a considerably wider range of drug sensitivities than our HNSCC lines.

Significant correlations were found between the tumour responses to cisplatin treatment in mice and the AUC of total platinum levels present in the xenografts. Such a significant correlation was also found for the corresponding cultured cells (Welters et al, 1997*a*). Although the ranking of the sensitivities to cisplatin of the HNSCC tumour lines and of the corresponding cell lines did not correlate, the total platinum levels in both cases did correlate with the sensitivity. Johnsson and Cavallin-Ståhl (1996) showed that the platinum distribution within squamous cell carcinoma tumours was fairly homogeneous, thus cisplatin was capable of penetrating into the whole tumour. From this observation and our results, it can be concluded that the tumour (cell) response is reflected by the total platinum levels in the tumour cells.

In vitro, a relationship was established between cisplatin sensitivity of the cultured cells and the level of the major intrastrand cross-link, platinum–AG. This correlation was only found at 1 h after the start of the treatment, suggesting that the level of DNA damage immediately after cisplatin incubation is very important for the efficacy of the treatment. However, in our panel of HNSCC lines, no significant correlation was observed with the main adduct, platinum–GG. Also, in the in vivo study, a significant correlation was found between the sensitivity of the tumour and the platinum–AG adducts. For this correlation, the AUC over the 3 h to 3 days interval after the second cisplatin treatment of the mice was used, a time period during which the adduct levels reached a maximum. After 3 days, these levels declined with time. This finding was consistent with results described by Poirier and co-workers (1992), who reported an increase in adduct levels in various rat tissues between 4 h and 2 days after single and multiple i.v. injection(s) of cisplatin followed by a decrease between days 2 and 7 and stable levels until 14 days. The AUC reflects the net result of DNA adduct formation and DNA repair. The importance of applying this AUC, derived from the platinum–DNA adduct level vs time plot, has been emphasized by Schellens et al (1996), who showed a correlation with the tumour response of this AUC in WBC of patients. This parameter, calculated over a period of 15 h after a 3-h cisplatin infusion, appeared to have a predictive value for the tumour response of patients receiving daily treatments. The fact that in the present study significant correlations were found between the platinum–AG, but not the platinum–GG, adduct levels and the HNSCC response suggests that platinum–AG plays an important role in the anti-tumour effect in the HNSCC lines studied. This finding is in agreement with the results of Fichtinger-Schepman et al (1995*b*). These authors determined the quantities of the various platinum–DNA adducts after treatment of Chinese hamster ovary cells with equitoxic doses of cisplatin and carboplatin, and provided evidence that platinum–AG may be the cytotoxic lesion. The platinum–AG adduct has also been found to be more mutagenic than platinum–GG (Burnouf et al, 1987; Yarema et al, 1995) and it is more effectively repaired in a cisplatin-resistant human testicular teratoma cell line (Hill et al, 1994). Los and coworkers (1995) reported that the ratio of platinum–DNA adducts to total platinum levels may be informative for the response of the tumour to cisplatin therapy. They suggested that a low ratio was indicative of high cellular detoxification due to interaction with glutathione and metallothioneins. Calculation of these ratios for our panel of tumour lines revealed that RO had extremely high ratios at 3 h and 24 h after the cisplatin treatment (data not shown; cf. Figure 2). This suggests that in this xenograft a relatively large amount of the cisplatin is able to bind to cellular DNA, resulting in a high level of platinum–DNA adducts, which may explain the high cisplatin sensitivity of this tumour line. In the other tumour lines, the ratio of platinum–GG adducts to the total platinum content was about two- to tenfold lower, and in the case of platinum–AG adducts 7- to 17-fold (cf. Figure 2). Thus, by calculating the ratio of platinum–AG adduct to the platinum content, the in vivo sensitivity to cisplatin may be predicted. However, it is very important to sample at early time points after the start of therapy. This predictive value should be tested in more extensive studies. Because the pharmacokinetics of cisplatin have been reported to be very similar in nude mice and humans (Kubota et al, 1993), this ratio might also be very important for the prediction of tumour response in patients. In the present study, the in vitro sensitivity to cisplatin of HNSCC cells, however, was found not to be related to this ratio of platinum–DNA adducts to platinum content. This again is a discrepancy between the in vivo and in vitro situation, which suggests that cell culture systems are not reliable in predicting in vivo outcomes.

In conclusion, the different sensitivities to cisplatin in our panel of HNSCC lines were significantly correlated with the total platinum levels as well as with the platinum–AG adduct levels both in cultured cells (in vitro) and in the xenografts (in vivo). These data support previous evidence that platinum–AG may be the cytotoxic lesion. However, results obtained for the cultured cells are not predictive for the response to cisplatin of HNSCC tumours in nude mice.

### ACKNOWLEDGEMENTS

This study was financially supported by the Dutch Cancer Society (grant MBL 92-74). The authors thank JE Pankras, M Ooms and CJA van Moorsel for their technical assistance with the post-labelling assay.

© Cancer Research Campaign 1999

**REFERENCES**

Braakhuis BJM, Ruiz van Haperen VWT, Welters MJP and Peters GJ (1995) Schedule-dependent therapeutic efficacy of the combination of gemcitabine and cisplatin in head and neck cancer xenografts. *Eur J Cancer* **31A**: 2335–2340

Burnouf D, Daune M and Fuchs RPP (1987) Spectrum of cisplatin-induced mutations in *Escherichia coli*. *Proc Natl Acad Sci USA* **84**: 3758–3762

Carey TE, Wolf GT, Baker SR and Krause CJ (1990) Cell surface antigen expression and prognosis. In *Head and Neck Cancer*, *2*. Fee WE, Goepfert H, Johns ME and Ward PH (eds), pp. 77–82. BC Decker: Toronto

Eastman A (1986) Reevaluation of interaction of cis-dichloro(ethylenediammine)-platinum(II) with DNA. *Biochemistry* **25**: 3912–3915

Fichtinger-Schepman AMJ, Van der Veer JL, Den Hartog JHJ, Lohman PHM and Reedijk J (1985) Adducts of the antitumor drug cis-diamminedichloro-platinum(II) with DNA: formation, identification, and quantitation. *Biochemistry* **24**: 707–713

Fichtinger-Schepman AMJ, Van Oosterom AT, Lohman PHM and Berends F (1987) cis-Diamminedichloroplatinum(II)-induced DNA adducts in peripheral leukocytes from seven cancer patients: quantitative immunochemical detection of the adduct induction and removal after a single dose of cis-diamminodichloroplatinum(II). *Cancer Res* **47**: 3000–3004

Fichtinger-Schepman AMJ, Van der Velde-Visser SD, Van Dijk-Knijnenburg HCM, Van Oosterom AT, Baan RA and Berends F (1990) Kinetics of the formation and removal of cisplatin-DNA adducts in blood cells and tumor tissue of cancer patients receiving chemotherapy: comparison with in vitro adduct information. *Cancer Res* **50**: 7887–7894

Fichtinger-Schepman AMJ, Van Dijk-Knijnenburg HCM, Dijt FJ, Van der Velde-Visser SD, Berends F and Baan RA (1995*a*) Effects of thiourea and ammonium bicarbonate on the formation and stability of bifunctional cisplatin-DNA adducts: consequences for the accurate quantification of the adducts in (cellular) DNA. *J Inorg Biochem* **58**: 177–191

Fichtinger-Schepman AMJ, Van Dijk-Knijnenburg HCM, Van der Velde-Visser SD, Berends F and Baan RA (1995*b*) Cisplatin- and carboplatin-DNA adducts: is Pt-AG the cytotoxic lesion? *Carcinogenesis* **16**: 2447–2453

Fisch MJ, Howard KL, Einhorn LH and Sledge GW (1996) Relationship between platinum-DNA adducts in leukocytes of patients with advanced germ cell cancer and survival. *Clin Cancer Res* **2**: 1063–1066

Gupta-Burt S, Shamkhani H, Reed E, Tarone RE, Allegra CJ, Pai LH and Poirier MC (1993) Relationship between patient response in ovarian and breast cancer and platinum drug-DNA adduct formation. *Cancer Epidemiology Biomarkers* **2**: 229–234

Hill BT, Shellard SA, Fichtinger-Schepman AMJ, Schmoll HJ and Harstrick A (1994) Differential formation and enhanced removal of specific cisplatin-DNA adducts in two cisplatin-selected resistant human testicular teratoma sublines. *Anti-Cancer Drugs* **5**: 321–328

Johnsson A and Cavallin-Ståhl E (1996) A topographic study on the distribution of cisplatin in xenografted tumors on nude mice. *Anti-Cancer Drug* **7**: 70–77

Kelland LR, Jones M, Abel G, Valenti M, Gwynne J and Harrap KR (1992) Human ovarian-carcinoma cell lines and companion xenografts: a disease-oriented approach to new platinum anticancer drug discovery. *Cancer Chemother Pharmacol* **30**: 43–50

Kobayashi H, Man S, Graham CH, Kapitain SJ, Teicher BA and Kerbel RS (1993) Acquired multicellular-mediated resistance to alkylating agents in cancer. *Proc Natl Acad Sci USA* **90**: 3294–3298

Korst AEC, Van der Sterre MLT, Eeltink CM, Fichtinger-Schepman AMJ, Vermorken JB and Van der Vijgh WJF (1998) Pharmacokinetics of carboplatin with and without amifostine in patients with solid tumors. *Clin Cancer Res* (in press)

Kubota T, Inoue S, Furukawa T, Ishibiki K, Kitajima M, Kawamura E and Hoffman RM (1993) Similarity of serum-tumor pharmacokinetics of antitumor agents in man and mice. *Anticancer Res* **13**: 1481–1484

Los G, Blommaert FA, Barton R, Heath DD, Den Engelse L, Hanchett C, Vicario D, Weisman R, Thomas Robbins K and Howell SB (1995) Selective intra-artial infusion of high-dose cisplatin in patients with advanced head and neck cancer results in high tumor platinum concentrations and cisplatin-DNA adduct formation. *Cancer Chemother Pharmacol* **37**: 150–154

Motzer RJ, Reed E, Perera F, Tang D, Shamkhani H, Poirier MC, Tsai WY, Parker RJ and Bosl GJ (1994) Platinum-DNA adducts assayed in leukocytes of patients with germ cell tumors measured by atomic absorbance spectrometry and enzyme-linked immunosorbent assay. *Cancer* **73**: 2843–2852

Parker RJ, Dimery IW, Dabholkar M, Vionnet J and Reed E (1993) Platinum-DNA adduct in head and neck cancer patients receiving cisplatin and carboplatin chemotherapy. *Int J Oncol* **3**: 331–335

Pizao PE, Lyaruu DM, Peters GJ, Van Arke-Otte J, Winograd B, Giaccone G and Pinedo HM (1992) Growth, morphology and chemosensitivity studies on postconfluent cells cultured in ‘V’-bottomed microtiter plates. *Br J Cancer* **66**: 660–665

Poirier MC, Reed E, Litterst CL, Katz D and Gupta-Burt S (1992) Persistence of platinum-ammine-DNA adducts in gonads and kidneys of rats and multiple tissues from cancer patients. *Cancer Res* **52**: 149–153

Poirier MC, Reed E, Shamkhani H, Tarone RE and Gupta-Burt S (1993) Platinum drug–DNA interactions in human tissues measured by cisplatin-DNA enzyme-linked immunosorbent assay and atomic absorbance spectroscopy. *Environ Health Perspect* **99**: 149–154

Reed E, Ozols RF, Tarone R, Yupsa SH and Poirier MC (1988) The measurement of cisplatin-DNA adduct levels in testicular cancer patients. *Carcinogenesis* **9**: 1909–1911

Reed E, Parker RJ, Gill I, Bicher A, Dabholkar M, Vionnet JA, Bostick-Burton F, Tarone R and Muggia FM (1993) Platinum-DNA adduct in leukocyte DNA of a cohort of 49 patients with 24 different types of malignancies. *Cancer Res* **53**: 3694–3699

Schellens JHM, Ma J, Planting ASTh, Van der Burg MEL, Van Meerten E, De Boer-Dennert M, Schmitz PIM, Stoter G and Verweij J (1996) Relationship between the exposure to cisplatin, DNA-adduct formation in leukocytes and tumour response in patients with solid tumours. *Br J Cancer* **73**: 1569–1575

Siddik ZH, Boxall FE and Harrap KR (1987) Flameless atomic absorption spectrophotometric determination of platinum in tissues solubilized in hyamine hydroxide. *Anal Biochem* **163**: 21–26

Teicher BA, Herman TS, Holden SA, Wang YY, Pfeffer MR, Crawford JW and Frei E (1990) Tumor resistance to alkylating agents conferred by mechanisms operative only in vivo. *Science* **247**: 1457–1461

Tomayko M and Reynolds CP (1989) Determination of subcutaneous tumor size in athymic (nude) mice. *Cancer Chemother Pharmacol* **24**: 148–154

Van Dongen GAMS, Braakhuis BJM, Leyva A, Hendriks HR, Kipp BBA, Bagnay M and Snow GB (1989) Anti-tumor and differentiation-inducting activity of *N*, *N*-dimethylformamide (DMF) in head and neck cancer xenografs. *In J Cancer* **43**: 285–292

Welters MJP, Fichtinger-Schepman AMJ, Baan RA, Hermsen MAJA, Van der Vijgh WJF, Cloos J and Braakhuis BJM (1997*a*) Relationship between the parameters cellular differentiation, doubling time and platinum accumulation and cisplatin sensitivity in a panel of head and neck cancer cell lines. *Int J Cancer* **71**: 410–415

Welters MJP, Maliepaard M, Jacobs-Bergmans AJ, Baan RA, Schellens JHM, Ma J, Van der Vijgh WJF, Braakhuis BJM and Fichtinger-Schepman AMJ (1997*b*) Improved $^{32}$P-postlabelling assay for the quantification of the major platinum-DNA adducts. *Carcinogenesis* **18**: 1767–1774

Yarema KJ, Lippard SJ and Essigmann JM (1995) Mutagenic and genotoxic effects of DNA adducts formed by the anticancer drug *cis*-diamminedichloroplatinum(II). *Nucleic Acids Res* **23**: 4066–4072

© Cancer Research Campaign 1999
